# Release of endothelial activation markers in lungs of patients with malaria-associated acute respiratory distress syndrome

**DOI:** 10.1186/s12936-019-3040-3

**Published:** 2019-12-03

**Authors:** Thao-Thy Pham, Chuchard Punsawad, Supattra Glaharn, Simon F. De Meyer, Parnpen Viriyavejakul, Philippe E. Van den Steen

**Affiliations:** 1 Laboratory of Immunoparasitology, Department of Microbiology and Immunology, Rega Institute for Medical Research, KU Leuven, University of Leuven, Leuven, Belgium; 2 School of Medicine, Walailak University, Nakhon Si Thammarat, Thailand; 3 Department of Tropical Pathology, Faculty of Tropical Medicine, Mahidol University, Bangkok, Thailand; 4 Laboratory for Thrombosis Research, KU Leuven Campus Kulak Kortrijk, Kortrijk, Belgium

**Keywords:** MA-ARDS, *Plasmodium falciparum*, Endothelial activation, VWF, ANG-2, Lungs, Oedema, von Willebrand factor, Angiopoietin-2, Lung pathology

## Abstract

**Background:**

Malaria-associated acute respiratory distress syndrome (MA-ARDS) is an understudied complication of malaria and is characterized by pulmonary inflammation and disruption of the alveolar-capillary membrane. Its pathogenesis remains poorly understood. Since endothelial activation plays an important role in other malarial complications, the expression of two endothelial activation markers, von Willebrand factor (VWF) and angiopoietin-2 (ANG-2), was investigated in the lungs of patients with MA-ARDS.

**Methods:**

Post-mortem lung sections of *Plasmodium falciparum*-infected patients without alveolar oedema (NA), *P. falciparum*-infected patients with alveolar oedema (MA-ARDS), and uninfected people who died accidentally with no pathological changes to the lungs (CON) were immunohistochemically stained for VWF and ANG-2, and were evaluated with semi-quantitative analysis.

**Results:**

Alveolar oedematous VWF and ANG-2 and intravascular VWF staining were significantly increased in patients with MA-ARDS versus infected and uninfected control groups. The levels of VWF in the alveolar septa and endothelial lining of large blood vessels of patients with MA-ARDS was significantly decreased compared to controls. ANG-2 expression was increased in the alveolar septa of malaria patients without alveolar oedema versus control patients, while ANG-2^+^ leukocytes were increased in the alveoli in both infected patient groups.

**Conclusions:**

This study documents a high level of VWF and ANG-2, two endothelial activation markers in the oedematous alveoli of post-mortem lung sections of Thai patients with MA-ARDS. Decreased detection of VWF in the endothelial lining of blood vessels, in parallel with an increased presence of intravascular VWF staining suggests marked endothelial activation and Weibel–Palade body release in the lungs of patients with MA-ARDS.

## Background

The World Health Organization (WHO) malaria report described 219 million clinical cases and 435,000 deaths in 2017 [1]. Despite the availability of efficient anti-malarial treatment, transmission and severe disease of the *Plasmodium* parasite still occurs in 91 countries in sub-Saharan Africa, South-East Asia and South America [1]. A malaria infection can be asymptomatic or develop into mild disease, but a significant number of patients develop lethal complications. An understudied complication is malaria-associated acute respiratory distress syndrome (MA-ARDS). MA-ARDS may occur upon a *Plasmodium falciparum* or *Plasmodium vivax* infection. MA-ARDS is also one of the most prevalent complications in *Plasmodium knowlesi*-infected patients [2–4]. This lung complication mainly occurs in adults without pre-existing semi-immunity (e.g. travellers and residents of low transmission areas). MA-ARDS has a poor prognosis and leads to a lethality rate of up to 80%, despite anti-malarial treatment. In fact, MA-ARDS has been described to emerge at hospital admission and during anti-malarial treatment [2]. MA-ARDS is characterized by disruption of the alveolar-capillary membrane, which results into alveolar oedema and leads to a decreased gas exchange and hypoxaemia. Patients also present excessive inflammation, microhemorrhages and leukocyte extravasation in the lungs. Currently, the only available remedy is mechanical ventilation.

Endothelial activation plays an important role in severe malaria. During a *Plasmodium* infection, the vascular endothelium becomes activated through several pathogenic factors, such as sequestration, inflammatory cytokines and parasite constituents [5–7]. As a result, the endothelium immediately releases the contents of its Weibel–Palade bodies and induces upregulation of inflammatory molecules, which can further aggravate the disease. Biomarkers for endothelial activation, such as von Willebrand factor (VWF) and angiopoietin-2 (ANG-2), have been associated with disease severity and mortality in *Plasmodium* infections [8–11].

VWF is a multimeric glycoprotein that plays an important role in haemostasis, and is synthesized in endothelial cells (ECs) and megakaryocytes [12–14]. In ECs, VWF multimers are stored in Weibel–Palade bodies from which they are released into the circulation upon endothelial activation. After release, VWF multimers can stay anchored onto the endothelial lining, forming platelet-decorated VWF strings. Interestingly, VWF strings have been proposed to facilitate parasite sequestration, which can induce further inflammation and endothelial activation [15]. VWF-adhering platelets can for example bind to infected red blood cells (iRBCs) and bridge iRBCs to the endothelium, which may aid the parasite to evade splenic clearance [16, 17].

Endothelial activation also induces upregulation of ANG-2 and the release of Weibel–Palade body-stored ANG-2. ANG-2 is a glycoprotein that antagonizes the binding of ANG-1 to the tyrosine kinase receptor TIE-2 on ECs [18, 19]. While ANG-1/TIE-2 interactions maintain the quiescent state of the endothelium by inducing an anti-apoptotic and anti-inflammatory response, ANG-2 binding to TIE-2 prevents ANG-1 binding and increases the endothelial sensitivity for inflammation, coagulation and vascular permeability-inducing factors. Besides its suggested use as a plasma biomarker for disease severity in *P. falciparum* infections [20–25], ANG-2 was found on the vascular endothelium in brain sections of Vietnamese patients with cerebral malaria (CM) [26].

VWF and ANG-2 expression has not been investigated yet in patients with MA-ARDS. Therefore, the expression of these endothelial markers was investigated by immunohistochemical (IHC) analyses on lung sections of *P. falciparum*-infected patients without alveolar oedema, *P. falciparum*-infected patients with MA-ARDS and uninfected controls who died without any damage to the lungs. These data demonstrate a vast increase of VWF and ANG-2 in the alveoli of patients with MA-ARDS. This study comprises the first observations of endothelial activation markers in lungs of patients with MA-ARDS.

## Methods

### Patients

Post-mortem lungs from *P. falciparum*-infected Thai patients and controls were fixed with formalin and embedded in paraffin. These samples were obtained by The Department of Tropical Pathology (Faculty of Tropical Medicine, Mahidol University, Thailand) over a span of 30 years [27]. The available clinical data were compiled in Table 1. Samples were classified into three groups: (1) people who died accidentally with no pathological changes to the lungs as controls (CON, n = 9), (2) *P. falciparum*-infected patients without alveolar oedema (NA, n = 15) and (3) *P. falciparum*-infected patients with alveolar oedema (MA-ARDS, n = 13). Patients with severe malaria underwent antimalarial treatment with intravenous quinine. The study protocol was reviewed and approved by the Ethics Committee of Faculty of Tropical Medicine, Mahidol University (MUTM 2016-051-01 and MUTM 2016-051-02).

**Table 1:** Clinical characteristics of patients

	NA	MA-ARDS	N
			(NA − MA-ARDS)
Sex (male/female)	10/5	7/6	15−13
Age (years)	24.1 ± 3.9	30.0 ± 2.9	15−13
Days of hospitalization	4.2 ± 0.5	5.1 ± 0.6	9−10
Parasitaemia (iRBCs/µl)	538,420.5 ± 110 462.1	304,138.9 ± 124 790.7	13
Haemoglobin (g/dl)	10.6 ± 0.7	8.6 ± 0.7	13
Haematocrit (%)	33.1 ± 2.2	26.5 ± 2.2*	13
White blood cells (#/µl)	12,176 ± 2422	12,467 ± 2193	13−12
Cerebral malaria (%)	73	38	15−13
Shock (%)	20	0	15−13
Hepatic complications (%)	7	14	15−13
Renal complications (%)	0	31	15−13
Heart failure (%)	7	7	15−13

Data are expressed as mean ± SEM

*NA* no alveolar oedema, *MA-ARDS* malaria-associated acute respiratory distress syndrome

* Significant difference compared to NA, p < 0.05

### IHC staining

Lungs were compartmentalized in smaller pieces, embedded in paraffin and cut into 4 µm thick sections. Lung sections were deparaffinized by heating and rehydrated through graded concentrations of alcohol. Sections were washed with distilled water, followed by an antigen unmasking step according to manufacturer’s instructions (Antigen unmasking solution, Vector Laboratories, CA, USA). The following steps were all executed after a washing step with Tris buffered saline (pH 7.6) unless indicated otherwise. Sections were treated with 3% H_2_O_2_ [30 min at room temperature (RT) in the dark] to inactivate the endogenous peroxidase. Then, aspecific binding was blocked with goat serum (30 min at RT). The latter step was immediately followed by incubation with the primary rabbit polyclonal antibody for VWF (1/1000, ab6994, Abcam, Cambridge, UK) or ANG-2 (1/200, ab153934, Abcam) for 1 h at 37 °C. Afterwards, secondary antibody (rabbit IgG) was added for incubation (30 min at RT) and reacted with the avidin–biotin complex conjugated with horseradish peroxidase (Vectastain ABC Kit, Vector Laboratories) according to manufacturer’s instructions. The peroxidase staining was executed with the ImmPACT^®^ DAB peroxidase substrate Kit (Vector Laboratories). Sections were then washed with distilled water and counterstained with Mayer’s haematoxylin (Merck, Darmstadt, Germany). Finally, sections were dehydrated through graded concentrations of alcohol and mounted with a coverslip. For each patient, one lung section was IHC stained and analysed. Additionally, negative controls, i.e. serial sections that were stained without the primary antibody were analysed in parallel for each lung section (Additional files 1, 2, 3, 4).

### Semi-quantitative analysis of IHC lung sections

Whole images of IHC lung sections were scanned with a Nanozoomer (Hamamatsu Photonics, Herrsching am Ammersee, Germany) and pictures were taken at 5× and 20× magnification with the NDP viewer software (Hamamatsu Photonics). Ten random pictures were taken at 20× magnification for each IHC stained lung section and examined for the following parameters: percentage of alveoli with oedema, percentage of alveoli with ANG-2^+^ leukocytes, percentage of blood vessels with ANG-2 and VWF staining on the endothelial lining, percentage of blood vessels with intravascular VWF staining, percentage of blood vessels with ANG-2^+^ leukocytes, and ANG-2 and VWF staining intensity of alveolar septa and oedema. Ten pictures for each sample were scored in a range of 0–2 for ANG-2 and VWF staining intensity of alveolar septa and oedema, where 0 is no expression, 1 is moderate staining and 2 is strong staining. The relative score for the ANG-2 and VWF staining intensity of the alveolar septa was determined by the sum of the described scores for each picture. The total relative score for the alveolar oedematous ANG-2 and VWF staining was determined by multiplying the fraction of alveoli with positively ANG-2 or VWF stained oedema, with the described intensity score for each picture, and this was summed up for the ten pictures of each sample.

### Statistics

The Mann–Whitney U test was used to determine the statistical significance of differences. Statistical analysis was done using the GraphPad Prism software (GraphPad software, San Diego, USA). P-values smaller than 0.05 were considered statistically significant. P-values were defined as follows: *p < 0.05, **p < 0.01, ***p < 0.001, ****p < 0.0001. Each dot represents the results from an individual person. Horizontal lines represent group medians. Asterisks without horizontal lines represent significant differences compared to the uninfected control group (CON). Horizontal lines with asterisk on top indicate pairwise significant differences between patient groups.

## Results

### Clinical data of patients

Thirty-seven patients were included in this study. The clinical characteristics of *P. falciparum*-infected patients are described in Table 1. There was no significant difference in age, days of hospitalization, parasitaemia, haemoglobin levels and circulating white blood cells between the NA and MA-ARDS group. A small decrease in haematocrit values was found in the MA-ARDS group compared to the NA group. Details of diagnosis are described in Table 1. Histopathological findings of these samples were previously described by Punsawad et al. [27].

### Expression of VWF in lungs of patients with MA-ARDS

**Fig. 1:**
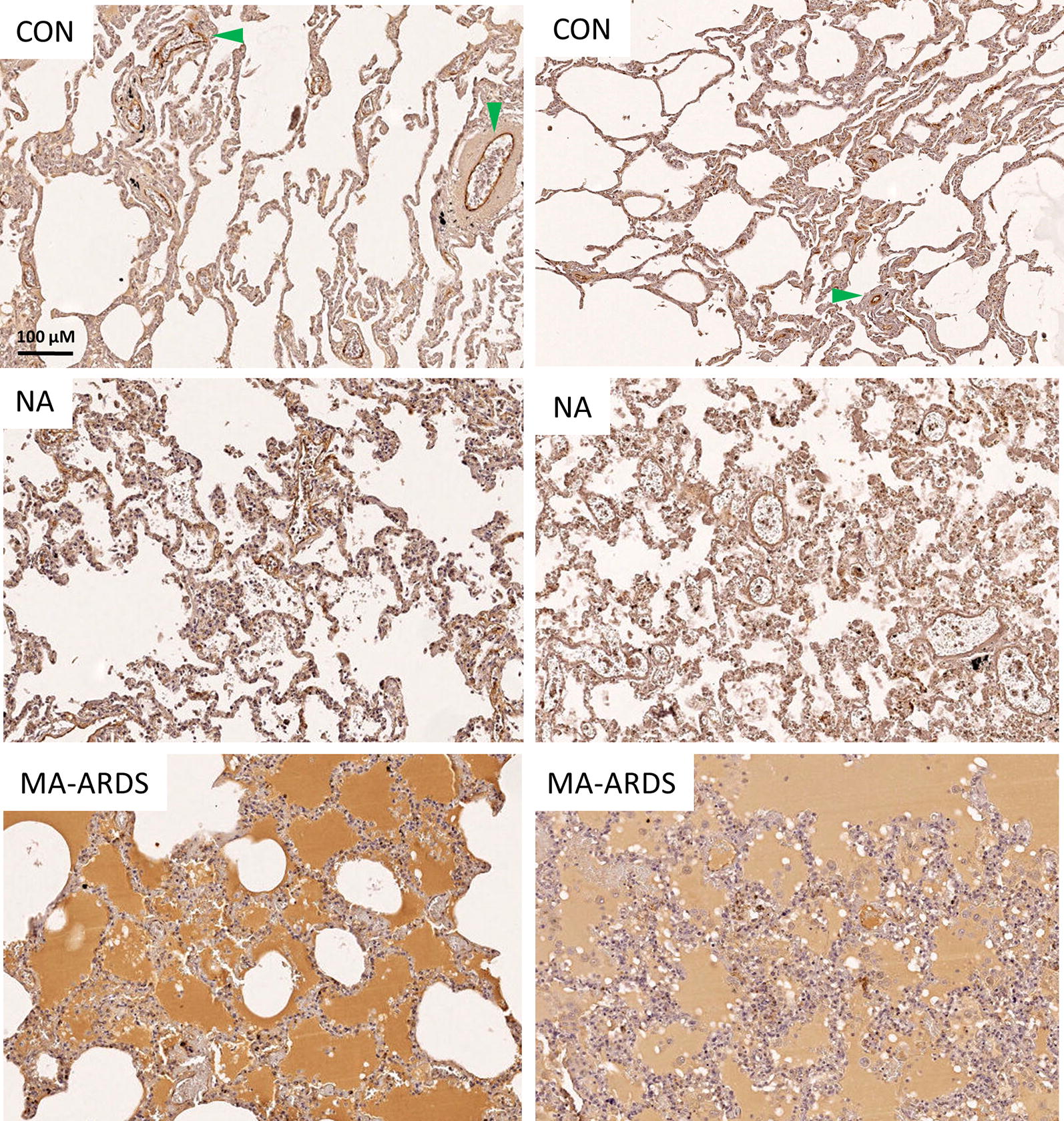
IHC staining of VWF in lungs of *P. falciparum*-infected patients and control group. VWF staining was executed in lung sections of people that died suddenly without any lung damage (CON), *P. falciparum*-infected patients without alveolar oedema (NA) and *P. falciparum*-infected patients with alveolar oedema (MA-ARDS). Each panel shows a section from a different patient. Green arrowheads indicate VWF stained large blood vessels. All images were taken at ×5 magnification. Bar = 100 µM

**Fig. 2:**
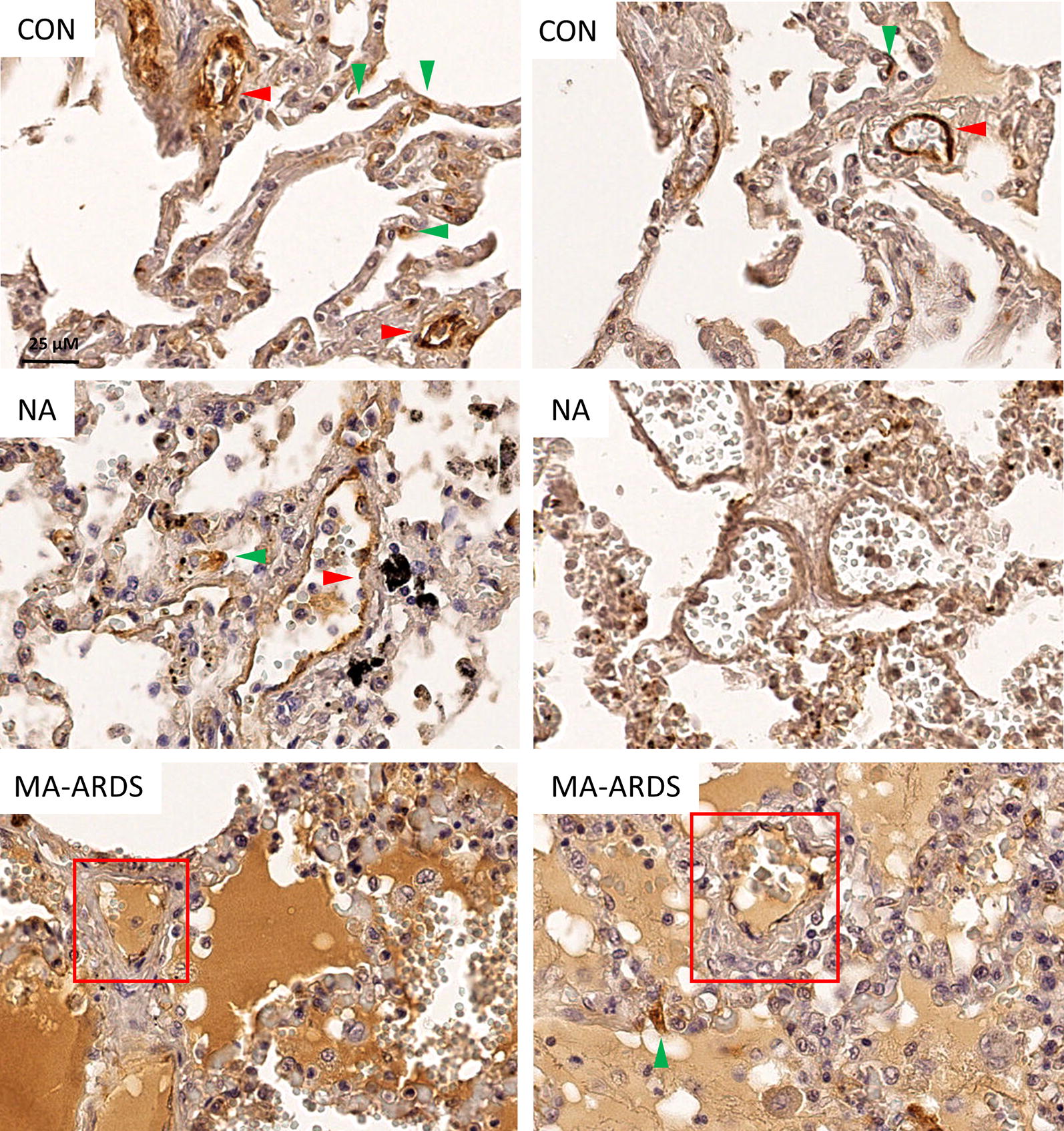
Expression of VWF in lungs of *P. falciparum*-infected patients and control group. VWF staining was executed in lung sections of people that died suddenly without any lung damage (CON), *P. falciparum*-infected patients without alveolar oedema (NA) and *P. falciparum*-infected patients with alveolar oedema (MA-ARDS). Each panel shows a section from a different patient. Green arrowheads indicate VWF staining in the alveolar septa. Red arrowheads indicate larger VWF stained blood vessel walls. Red frames demonstrate intravascular VWF staining. All images were taken at ×20 magnification. Bar = 25 µM

Since circulating VWF is correlated with disease severity in *P. falciparum*-infected patients, the expression of VWF in MA-ARDS was investigated [10, 11, 28, 29]. Lung sections of people that died suddenly without any lung damage (CON), *P. falciparum*-infected patients without alveolar oedema (NA) and *P. falciparum*-infected patients with alveolar oedema (MA-ARDS) were stained with IHC for the early endothelial activation marker VWF. A broad view of these lung sections demonstrated strong VWF expression on the endothelial lining of large blood vessels in control samples (Fig. 1). Strong VWF staining was also observed in alveolar oedema of patients with MA-ARDS. A more detailed view showed that the VWF immunoreactivity is present on the endothelial lining of large blood vessels, alveolar septa and alveolar oedema (Fig. 2). Several large blood vessels showed VWF staining in the lumen, suggesting thrombus-like intravascular VWF staining (Fig. 2).

**Fig. 3:**
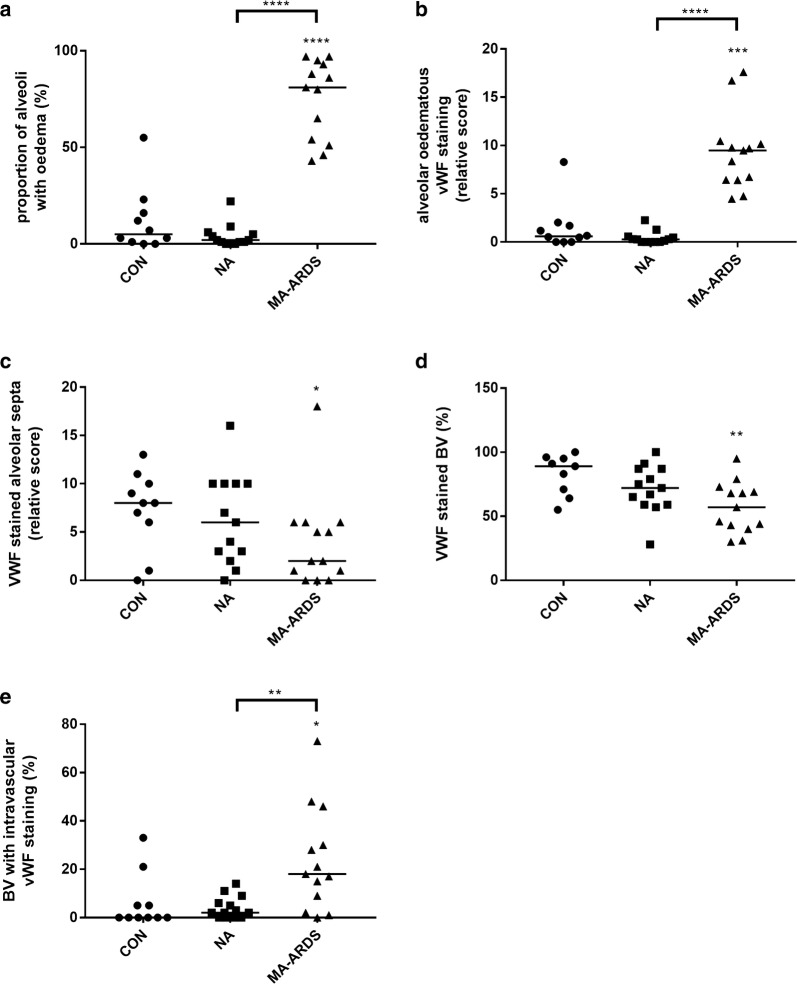
Semi-quantitative analysis of VWF expression in lungs of *P. falciparum*-infected patients and control group. VWF staining was executed in lung sections of people that died suddenly without any lung damage (CON), *P. falciparum*-infected patients without alveolar oedema (NA) and *P. falciparum*-infected patients with alveolar oedema (MA-ARDS). **a** The incidence in oedema was determined. Subsequently, semi-quantitative staining was performed for the following parameters: **b** alveolar oedematous VWF staining, **c** VWF stained alveolar septa, **d** large blood vessels with VWF stained endothelial lining and **e** intravascular VWF staining. N = 9–13

Semi-quantitative analyses demonstrated a significantly higher incidence of alveolar oedema in patients with MA-ARDS compared to the CON and NA group (Fig. 3a). This was paralleled by a significant increase in VWF stained alveolar oedema in the MA-ARDS group (Fig. 3b). The alveolar oedema varied in VWF staining intensity (Figs. 1, 2), and this was taken in consideration during the scoring for the semi-quantitative analysis. Patients with MA-ARDS also showed significantly decreased VWF expression in alveolar septa and large blood vessel walls compared to uninfected controls (Fig. 3c, d). In contrast, the incidence of intravascular VWF staining was increased in the MA-ARDS group and was significantly different from the CON and NA group (Fig. 3e). No difference was observed between the NA and CON group for the mentioned parameters. Overall, these data demonstrate a vastly increased intraluminal VWF staining in alveoli and large blood vessels in lungs of patients with MA-ARDS.

### IHC analysis of ANG-2 expression in MA-ARDS

**Fig. 4:**
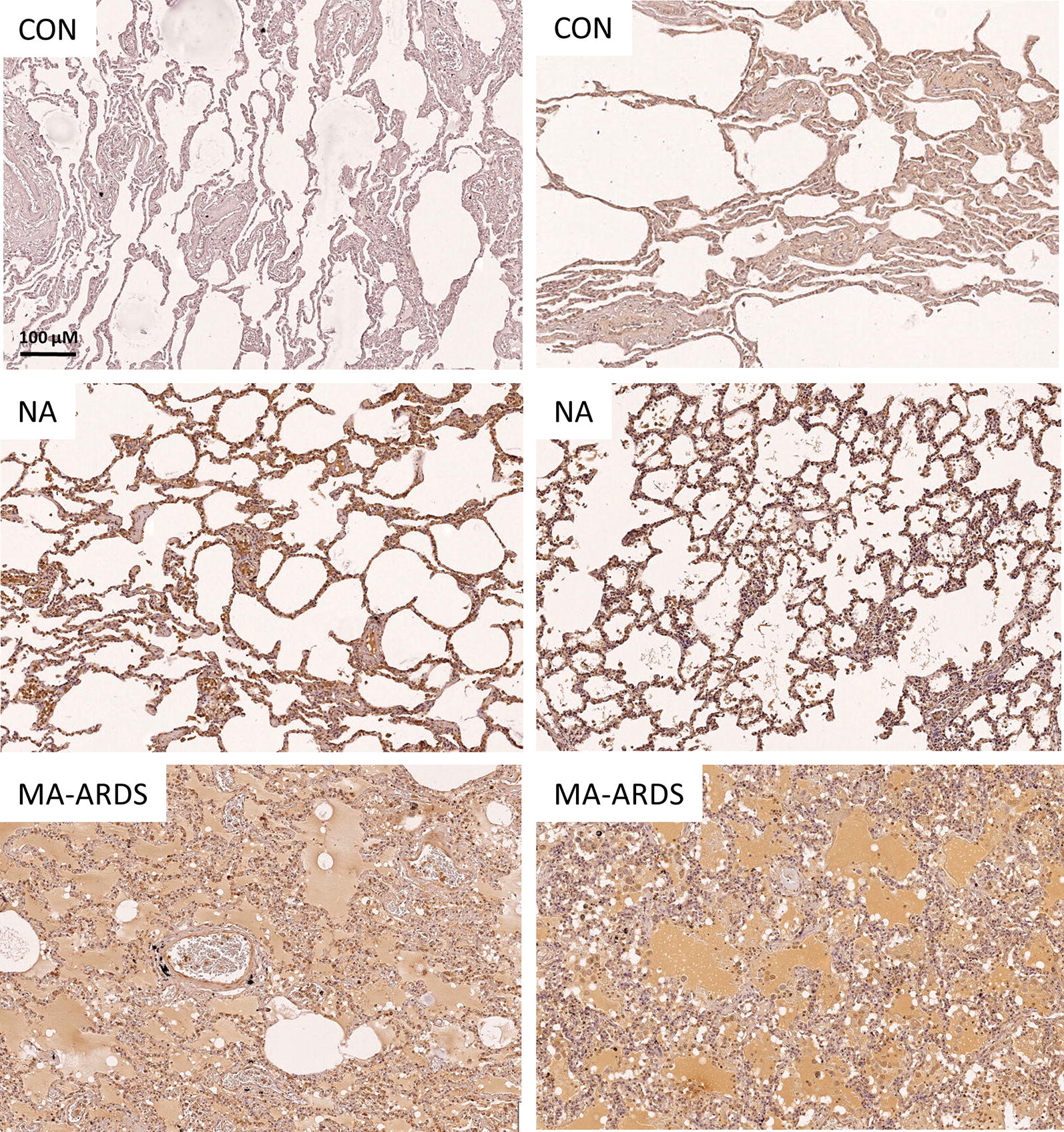
IHC staining of ANG-2 in lungs of *P. falciparum*-infected patients and control group. ANG-2 staining was executed in lung sections of people that died suddenly without any lung damage (CON), *P. falciparum*-infected patients without alveolar oedema (NA) and *P. falciparum*-infected patients with alveolar oedema (MA-ARDS). Each panel shows a section from a different patient. All images were taken at ×5 magnification. Bar = 100 µM

**Fig. 5:**
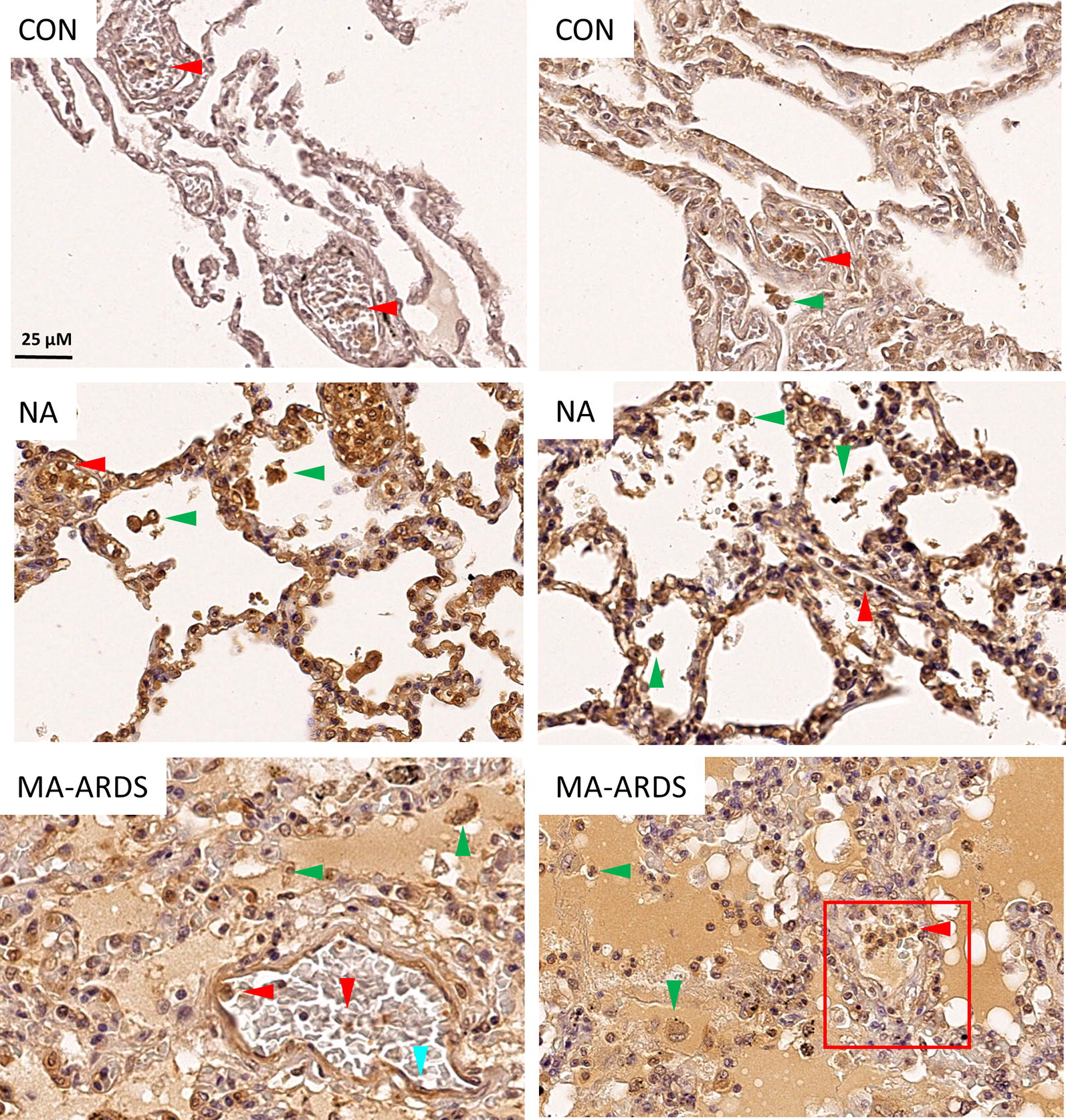
Expression of ANG-2 in lungs of *P. falciparum*-infected patients and control group. ANG-2 staining was executed in lung sections of people that died suddenly without any lung damage (CON), *P. falciparum*-infected patients without alveolar oedema (NA) and *P. falciparum*-infected patients with alveolar oedema (MA-ARDS). Each panel shows a section from a different patient. Arrowheads indicate ANG-2 expression in endothelial lining of large blood vessels (blue), and ANG-2^+^ leukocytes in alveoli (green) and in large blood vessels (red). Red frames demonstrate intravascular ANG-2 staining. All images were taken at ×20 magnification. Bar = 25 µM

Endothelial activation in MA-ARDS was further investigated by determining the pulmonary expression of ANG-2. ANG-2 is an endothelial activation marker and has also been used as a biomarker for disease severity in *P. falciparum* infections [20–24]. Figure 4 demonstrates an extensively increased expression of ANG-2 in the alveoli of patients with MA-ARDS compared to the CON and NA group. ANG-2 expression was more specifically found in alveolar oedema, alveolar septa, endothelial lining and lumen of large blood vessels, and leukocytes in large blood vessels and alveoli (Fig. 5).

**Fig. 6:**
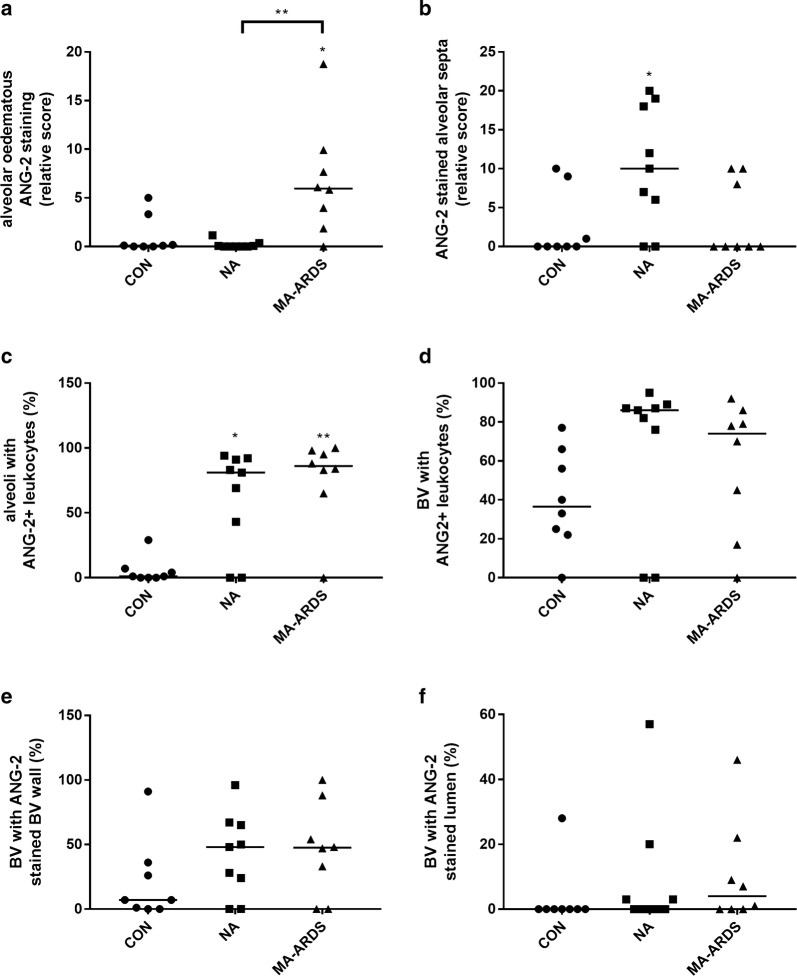
Semi-quantitative analysis of ANG-2 expression in lungs of *P. falciparum*-infected patients and control group. ANG-2 staining was executed in lung sections of people that died suddenly without any lung damage (CON), *P. falciparum*-infected patients without alveolar oedema (NA) and *P. falciparum*-infected patients with alveolar oedema (MA-ARDS). Semi-quantitative staining was performed for the following parameters: **a** alveolar oedematous ANG-2 staining, **b** ANG-2 stained alveolar septa, **c**, **d** alveoli and large blood vessels with ANG-2^+^ leukocytes, **e** large blood vessels with ANG-2 stained endothelial lining and **f** large blood vessels with ANG-2 stained lumen. N = 8–9

The variability in the intensity of ANG-2 expression in alveolar oedema was taken in consideration during the scoring. The semi-quantitative analysis showed a significant increase in oedematous ANG-2 staining in patients with MA-ARDS compared to the CON and NA group (Fig. 6a). While patients with MA-ARDS had similar ANG-2 expression in the alveolar septa, *P. falciparum*-infected patients without alveolar oedema had a significant increase versus the CON group (Fig. 6b). A higher amount of ANG-2^+^ leukocytes was observed in both *P. falciparum*-infected patient groups (Fig. 6c). Additionally, a non-significant trend was found in *P. falciparum*-infected patients without alveolar oedema for increased circulating ANG-2^+^ leukocytes compared to CON groups (p = 0.0556) (Fig. 6d). The ANG-2 staining on the endothelial lining and in the lumen of large blood vessels did not differ in between the investigated patient groups (Fig. 6e, f).

Overall, these data show that ANG-2 staining is increased in lungs of malaria patients versus controls, with a marked staining of the alveolar septa in the NA group and an extensive increase of oedematous ANG-2 staining in the alveoli of patients with MA-ARDS.

## Discussion

This study documents an extensive increase of VWF and ANG-2, two endothelial activation markers in the alveoli of post-mortem lung sections of Thai patients with MA-ARDS. VWF and ANG-2 stained alveolar oedema and intravascular VWF staining were significantly increased in patients with MA-ARDS versus infected and uninfected control groups. The staining of VWF on the alveolar septa and endothelial lining of large blood vessels of patients with MA-ARDS was significantly decreased compared to controls. The staining of ANG-2 was increased on the alveolar septa in infected patients without alveolar oedema versus control patients, while ANG-2^+^ leukocytes were increased in the alveoli in both infected patient groups.

Histopathological findings of these samples have been described in an earlier study [27]. Haematoxylin–eosin staining of lung sections of patients with MA-ARDS demonstrated alveolar oedema, vast infiltration of leukocytes and haemorrhages. This post-mortem study also showed presence of iRBCs in the blood vessels, suggesting parasite sequestration in the lungs [27]. Samples of patients with MA-ARDS were compared with *P. falciparum*-infected patients without alveolar oedema and uninfected people that died suddenly without any damage to the lungs. These three patient groups are suited to investigate the expression of endothelial activation markers in MA-ARDS.

VWF is released early into the circulation upon infection with *P. falciparum* infection [30]. Higher VWF antigen and VWF propeptide plasma values were found in patients infected with *P. falciparum* compared to healthy controls [23, 29, 31–33]. Moreover, a correlation between circulating VWF and disease severity was observed in *P. falciparum*-infected patients [10, 11, 28], but conflicting results were also published [29]. A Disintegrin and Metalloproteinase with a ThromboSpondin type 1 motif, member 13 (ADAMTS13) cleaves the ultra large-VWF multimer in smaller less reactive multimers. ADAMTS13 plasma levels and activity were decreased in *Plasmodium*-infected patients [28, 32, 34, 35]. This decrease in ADAMTS13 plasma levels and activity correlated with disease severity.

Patient data were validated in a mouse model for CM i.e. the infection of *Plasmodium berghei* (ANKA strain) in C57BL/6 mice. These mice develop experimental CM with increased VWF antigen plasma levels, ultra large-VWF multimers and VWF activity [36]. However, ADAMTS13 activity stayed unchanged. Kraisin et al. investigated VWF in the experimental MA-ARDS model, i.e. the infection of *P. berghei* (NK65-E strain) in C57BL/6 mice [37–39]. Similar to patient data, they found increased VWF levels and reduced ADAMTS13 activity levels in plasma of infected mice. Infected VWF KO developed less alveolar oedema versus WT mice upon infection. However, these infected mice still died earlier, possibly due to an increased parasite load in the circulation and the lungs [37]. Despite this difference in survival, the study does propose a pathogenic role of VWF on the integrity of the endothelial barrier.

These data showed a significant increase of VWF stained alveolar oedema and intravascular VWF staining in patients with MA-ARDS. VWF stained alveolar oedema may be the result of circulating VWF leaking into the alveoli due to the disruption of the alveolar-capillary membrane. The intravascular staining is not observed in the control group, suggesting that it is not the result of an isolation artefact. The increased incidence of intravascular VWF staining together with the decreased immunoreactivity of VWF in the endothelial lining of large blood vessels in the MA-ARDS group strongly suggest endothelial activation with a massive exocytosis of the Weibel–Palade bodies.

In lungs of healthy controls, strong VWF staining is present on the endothelial lining of larger blood vessels, however the capillary endothelium has been described to be mostly unreactive for VWF [40–42]. This VWF staining intensity was also described to increase with vessel size [40, 43]. These data demonstrated VWF staining in the alveolar septa (Fig. 2), which may be ascribed to the few septal capillaries that have positive VWF immunoreactivity. Additionally, pulmonary circulating megakaryocytes, which constitutively express VWF, have been found to generate 50% of the platelet reservoir in mice [44, 45]. Thrombocytopaenia is one of the most common haematological complications in malaria infections and studies have proposed dysmegakaryopoiesis to be one of the mechanisms behind this haematological disturbance in malaria [46, 47]. The decreased VWF staining in the alveolar septa of patients with MA-ARDS versus the control group may possibly be due to an even more decreased VWF staining of capillaries and/or a decreased expression of VWF in circulating megakaryocytes in the lungs.

Another commonly investigated endothelial activation marker is ANG-2. In line with the meta-analysis of De Jong et al. [48], an increased ANG-2/ANG-1 ratio and a decreased ANG-1 may be used as a biomarker for disease severity in *P. falciparum* infections, and seems to be prominent in cerebral complications [20–24]. However, ANG-2 plasma levels was also used alone as a biomarker [49–51]. ANG-2 plasma levels remain high after anti-malarial treatment in paediatric patients with CM, suggesting a persistent endothelial activation [51].

A protective effect of ANG-1 has been shown in the mouse model of experimental CM [24]. ANG-2, which competitively antagonizes the TIE-2 receptor for ANG-1 binding, may therefore aggravate the disease. Kim et al. showed that on 5 days post-infection (dpi), ANG-2 expression in brains of *P. berghei* (ANKA strain)-infected C57BL/6 mice was increased compared to uninfected mice [52]. The limited data regarding the ANG/TIE-2 pathway in experimental malaria models, illustrate the need for more studies.

A significant increase of ANG-2 staining in patients with MA-ARDS was demonstrated through increased alveolar oedematous ANG-2 staining. The presence of ANG-2 in the alveolar oedema may also be due to a vascular leakage into the alveoli. ANG-2^+^ leukocytes may further increase the ANG-2 levels in oedematous alveoli through secretion of ANG-2. ANG-2 expression in the alveolar septa was increased in *P. falciparum*-infected patients without alveolar oedema, but not in patients with MA-ARDS, suggesting a different ANG/TIE-2 pathway regulation between infected groups.

An inherent limitation of this post-mortem study is the anti-malarial treatment of these hospitalized patients. Upon admission and diagnosis, patients started a seven-day treatment scheme of intravenous quinine, but died on average 4–5 days after hospitalization. It cannot be excluded that the intravenous quinine treatment affected the development of MA-ARDS and/or endothelial activation in these patients. Nevertheless, the data clearly show increased pulmonary VWF and ANG-2 staining in post-mortem MA-ARDS samples, suggesting massive endothelial activation.

The increase of VWF and ANG-2 may not be specific for MA-ARDS since endothelial activation is also prominently present in non-malarial ARDS [53]. Several clinical studies have shown a prognostic value for circulating VWF and ANG-2 in these patients [54–57]. However, conflicting data have been found [55–60]. While Attia et al. did not observe a difference in ANG-2 levels in bronchoalveolar lavage fluid of patients with ARDS versus healthy controls, the research group of Ando et al. did demonstrate a significant increase of ANG-2 in patients with ARDS versus patients with idiopathic pulmonary fibrosis [57, 61]. These inconsistencies may be ascribed to multiple factors, such as variable ARDS severities and different non-malarial ARDS pathologies (e.g. direct versus indirect lung injury). Due to limited data, more studies including post-mortem analyses, are suggested to obtain a better view on the expression of endothelial activation markers in lungs of patients with non-malarial ARDS.

## Conclusions

In conclusion, these data show a high level of VWF and ANG-2 in the oedematous alveoli of post-mortem lung sections of Thai patients with MA-ARDS. The decreased detection of VWF in the endothelial lining of large blood vessels and increased presence of intravascular VWF staining suggest an extensive endothelial activation and the content release of the Weibel–Palade bodies. This study demonstrates the first findings for the endothelial activation markers VWF and ANG-2 in lungs of patients with MA-ARDS. Therefore, more studies to fully understand the VWF and ANG/TIE-2 axis in MA-ARDS are warranted.

## Supplementary information

**Additional file 1.** Negative controls of IHC staining of VWF in lungs of *P. falciparum*-infected patients and control group. Serial lung sections of people that died suddenly without any lung damage (CON), *P. falciparum*-infected patients without alveolar oedema (NA) and *P. falciparum*-infected patients with alveolar oedema (MA-ARDS) were stained in parallel without the primary antibody for VWF. Each panel demonstrates the complementary negative control for the sections in Fig. 1. All images were taken at 5x magnification. Bar = 100 µM.

**Additional file 2.** Negative controls of IHC lung sections for VWF in lungs of *P. falciparum*-infected patients and control group. Serial lung sections of people that died suddenly without any lung damage (CON), *P. falciparum*-infected patients without alveolar oedema (NA) and *P. falciparum*-infected patients with alveolar oedema (MA-ARDS) were stained in parallel without the primary antibody for VWF. Each panel demonstrates the complementary negative control for the sections in Fig. 2. All images were taken at 20x magnification. Bar = 25 µM.

**Additional file 3.** Negative controls of IHC staining of ANG-2 in lungs of *P. falciparum*-infected patients and control group. Serial lung sections of people that died suddenly without any lung damage (CON), *P. falciparum*-infected patients without alveolar oedema (NA) and *P. falciparum*-infected patients with alveolar oedema (MA-ARDS) were stained in parallel without the primary antibody for ANG-2. Each panel demonstrates the complementary negative control for the sections in Fig. 4. All images were taken at 5x magnification. Bar = 100 µM.

**Additional file 4.** Negative controls of IHC lung sections for ANG-2 in lungs of *P. falciparum*-infected patients and control group. Serial lung sections of people that died suddenly without any lung damage (CON), *P. falciparum*-infected patients without alveolar oedema (NA) and *P. falciparum*-infected patients with alveolar oedema (MA-ARDS) were stained in parallel without the primary antibody for ANG-2. Each panel demonstrates the complementary negative control for the sections in Fig. 5. All images were taken at 20x magnification. Bar = 25 µM.

## Data Availability

All data generated or analysed during this study are included in this published article (and its additional information files).

## Abbreviations

ADAMTS13: A Disintegrin and Metalloproteinase with a Thrombospondin type 1 motif, member 13
ANG-1: angiopoietin-1
ANG-2: angiopoietin-2
CM: cerebral malaria
CON: control
dpi: days post-infection
EC: endothelial cell
IHC: immunohistochemical
iRBC: infected red blood cell
MA-ARDS: malaria-associated acute respiratory distress syndrome
NA: no alveolar oedema
RT: room temperature
VWF: von Willebrand factor
