# Supplementary figures and images for: Release of endothelial activation markers in lungs of patients with malaria-associated acute respiratory distress syndrome
Source: Malar J. 2019 Dec 3;18:395. doi: 10.1186/s12936-019-3040-3 (PMC6891978; doi:10.1186/s12936-019-3040-3)

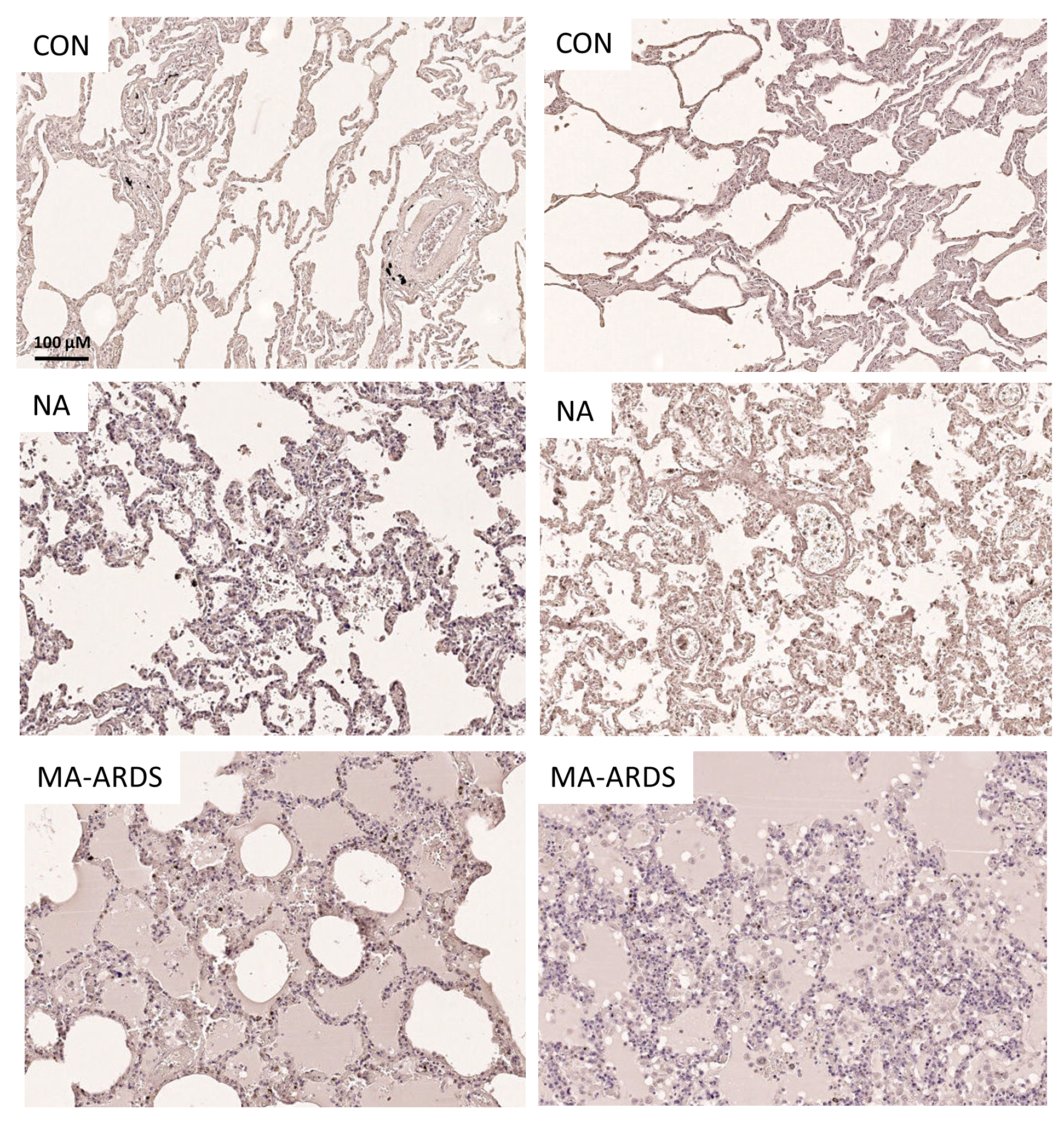

Supplement: Supplementary file 1 — Additional file 1. Negative controls of IHC staining of VWF in lungs of P. falciparum-infected patients and control group. Serial lung sections of people that died suddenly without any lung damage (CON), P. falciparum-infected patients without alveolar oedema (NA) and P. falciparum-infected patients with alveolar oedema (MA-ARDS) were stained in parallel without the primary antibody for VWF. Each panel demonstrates the complementary negative control for the sections in Fig. 1. All images were taken at 5x magnification. Bar = 100 µM. [file 12936_2019_3040_MOESM1_ESM.tif]

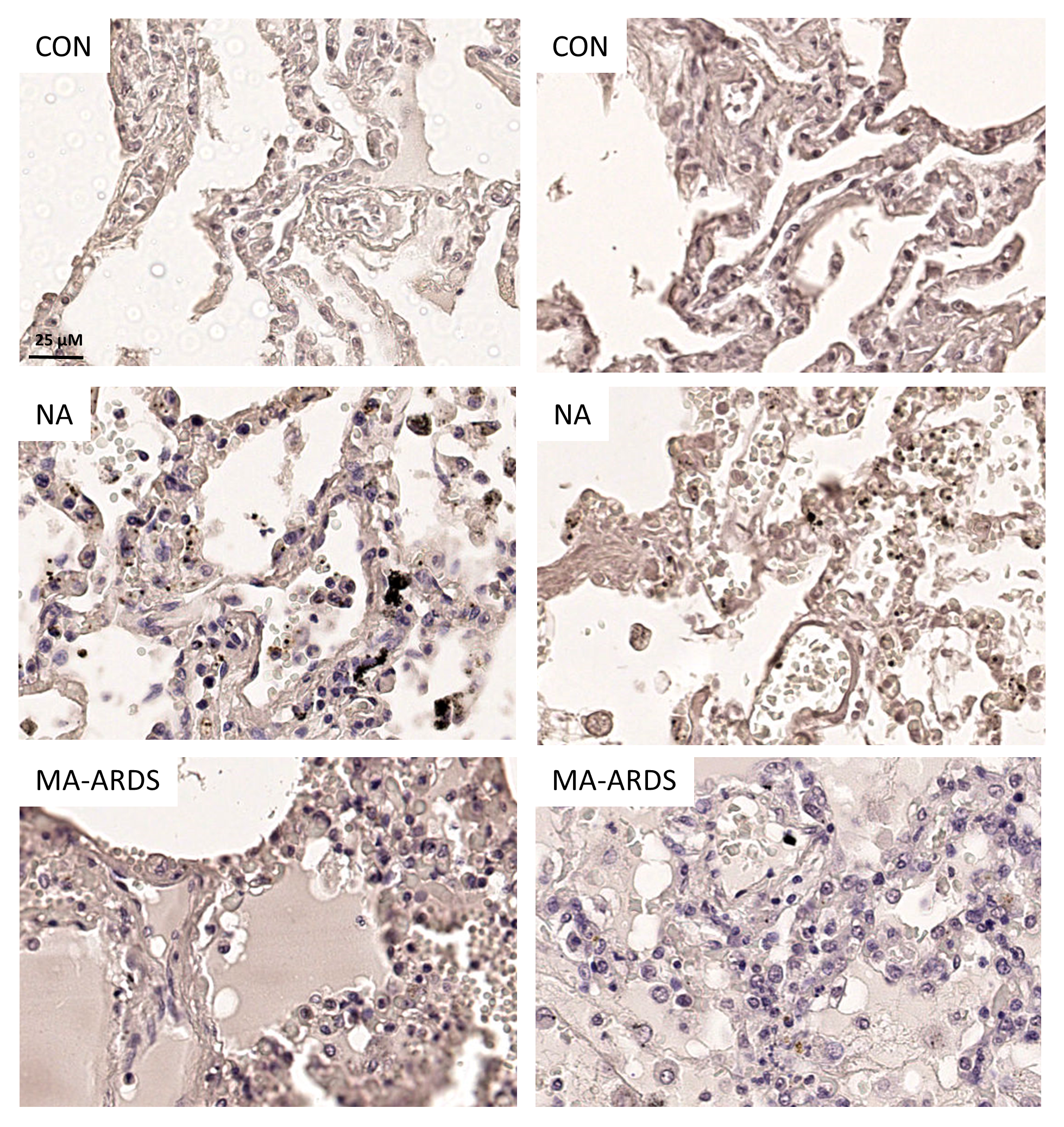

Supplement: Supplementary file 2 — Additional file 2. Negative controls of IHC lung sections for VWF in lungs of P. falciparum-infected patients and control group. Serial lung sections of people that died suddenly without any lung damage (CON), P. falciparum-infected patients without alveolar oedema (NA) and P. falciparum-infected patients with alveolar oedema (MA-ARDS) were stained in parallel without the primary antibody for VWF. Each panel demonstrates the complementary negative control for the sections in Fig. 2. All images were taken at 20x magnification. Bar = 25 µM. [file 12936_2019_3040_MOESM2_ESM.tif]

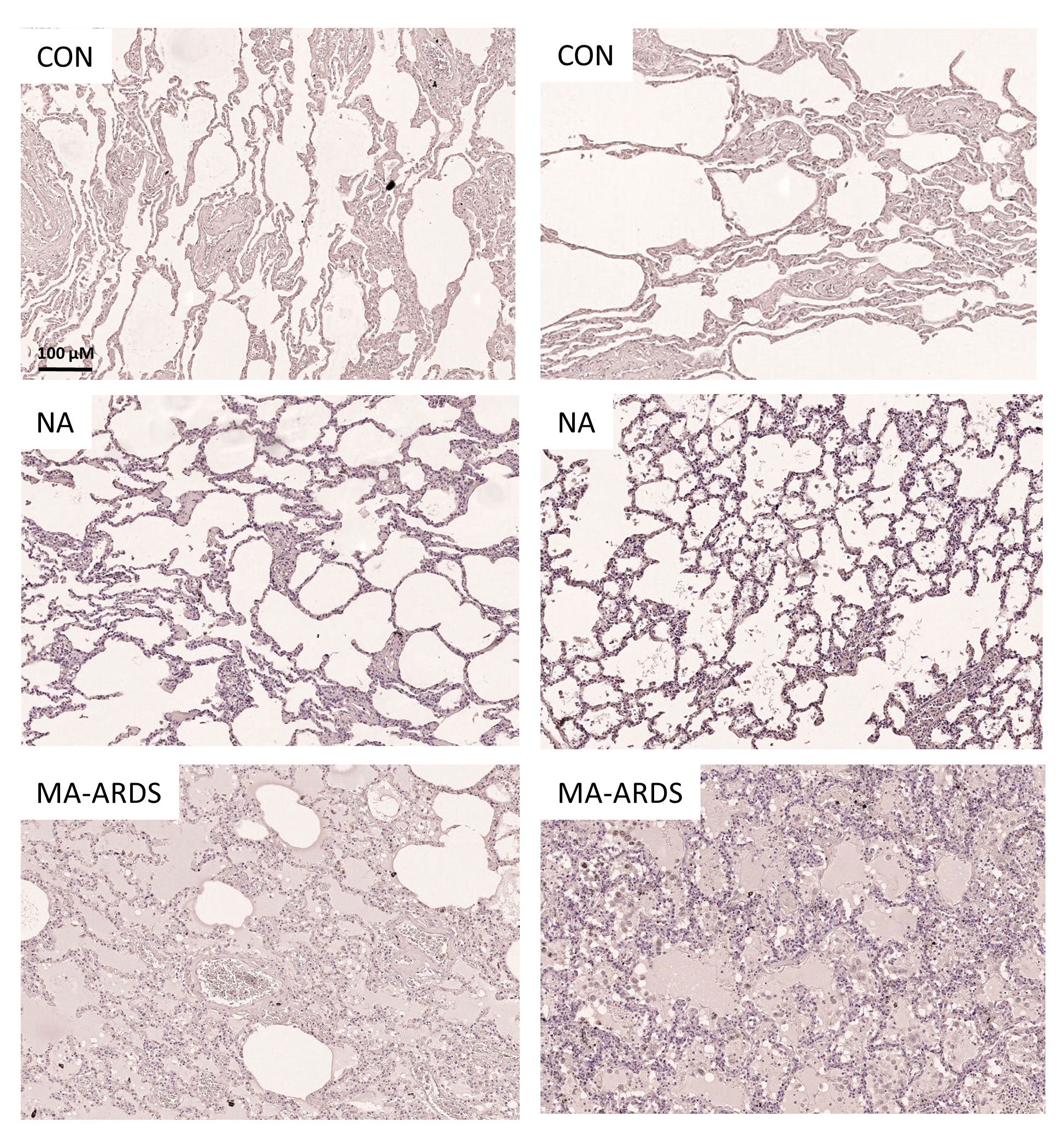

Supplement: Supplementary file 3 — Additional file 3. Negative controls of IHC staining of ANG-2 in lungs of P. falciparum-infected patients and control group. Serial lung sections of people that died suddenly without any lung damage (CON), P. falciparum-infected patients without alveolar oedema (NA) and P. falciparum-infected patients with alveolar oedema (MA-ARDS) were stained in parallel without the primary antibody for ANG-2. Each panel demonstrates the complementary negative control for the sections in Fig. 4. All images were taken at 5x magnification. Bar = 100 µM. [file 12936_2019_3040_MOESM3_ESM.tif]

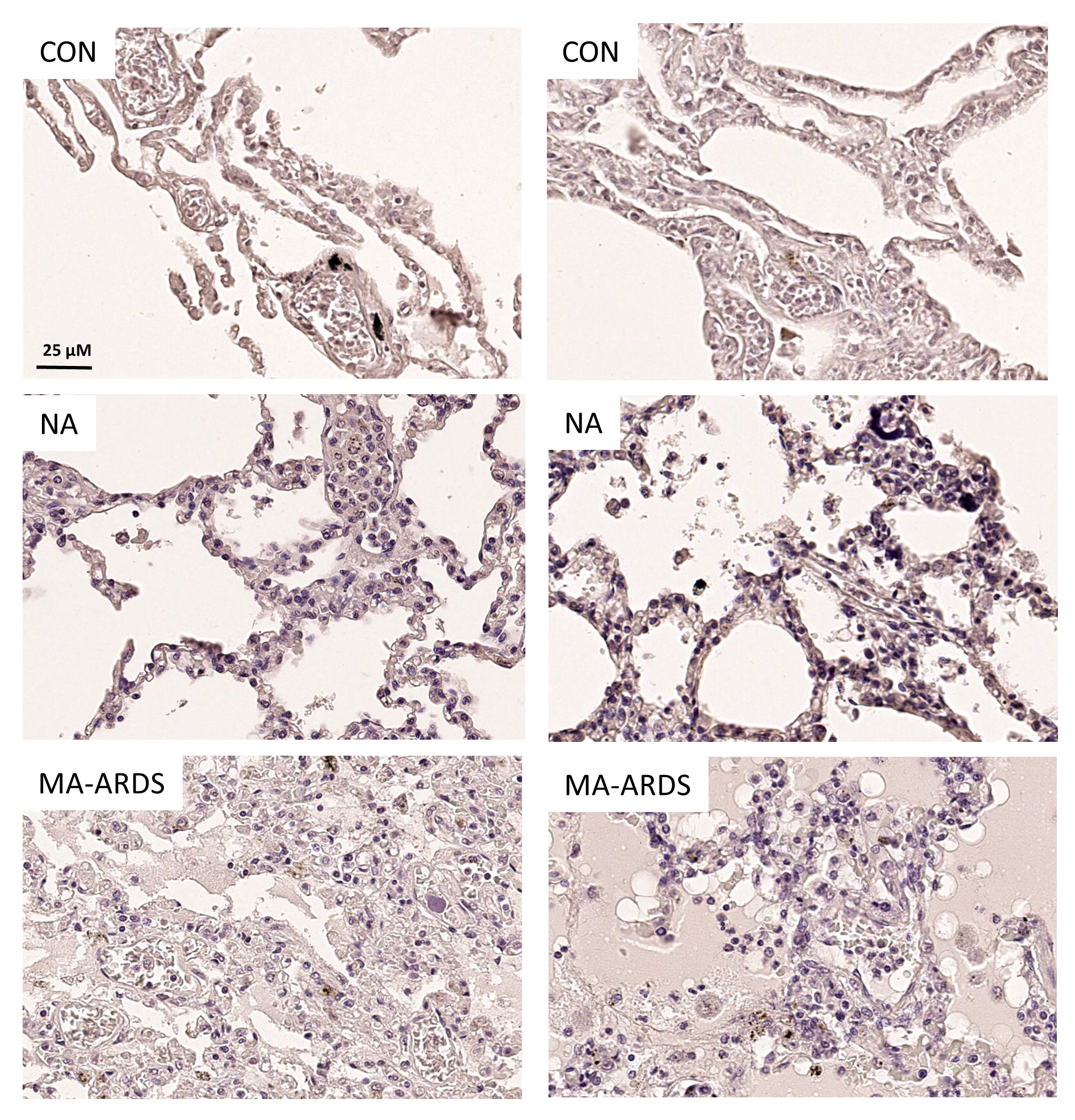

Supplement: Supplementary file 4 — Additional file 4. Negative controls of IHC lung sections for ANG-2 in lungs of P. falciparum-infected patients and control group. Serial lung sections of people that died suddenly without any lung damage (CON), P. falciparum-infected patients without alveolar oedema (NA) and P. falciparum-infected patients with alveolar oedema (MA-ARDS) were stained in parallel without the primary antibody for ANG-2. Each panel demonstrates the complementary negative control for the sections in Fig. 5. All images were taken at 20x magnification. Bar = 25 µM. [file 12936_2019_3040_MOESM4_ESM.tif]
